# Polypurine Reverse-Hoogsteen Hairpins as a Tool for Exon Skipping at the Genomic Level in Mammalian Cells

**DOI:** 10.3390/ijms22073784

**Published:** 2021-04-06

**Authors:** Véronique Noé, Carlos J. Ciudad

**Affiliations:** Department of Biochemistry and Physiology, School of Pharmacy and Food Sciences & IN2UB, University of Barcelona, 08028 Barcelona, Spain; cciudad@ub.edu

**Keywords:** exon skipping, PPRH, DHFR

## Abstract

Therapeutic strategies for rare diseases based on exon skipping are aimed at mediating the elimination of mutated exons and restoring the reading frame of the affected protein. We explored the capability of polypurine reverse-Hoogsteen hairpins (PPRHs) to cause exon skipping in NB6 cells carrying a duplication of exon 2 of the *DHFR* gene that causes a frameshift abolishing DHFR activity. Methods: Different editing PPRHs were designed and transfected in NB6 cells followed by incubation in a DHFR-selective medium lacking hypoxanthine and thymidine. Surviving colonies were analyzed by DNA sequencing, RT-PCR, Western blotting and DHFR enzymatic activity. Results: Transfection of editing PPRHs originated colonies in the DHFR-selective medium. DNA sequencing results proved that the *DHFR* sequence in all these colonies corresponded to the wildtype sequence with just one copy of exon 2. In the edited colonies, the skipping of the additional exon was confirmed at the mRNA level, the DHFR protein was restored, and it showed high levels of DHFR activity. Conclusions: Editing-PPRHs are able to cause exon skipping at the DNA level and could be applied as a possible therapeutic tool for rare diseases.

## 1. Introduction

Rare diseases are the result of point mutations in gene sequences that can alter the reading frame of the encoded protein or produce a premature stop codon. Exon skipping approaches can induce the skipping of specific exons bearing such mutations to restore the reading frame of the protein. This is the case for Duchenne muscular dystrophy (DMD), a degenerative muscle disease caused by mutations in the *DMD* gene encoding for the dystrophin protein, which causes an aberrant translation [1]. Since dystrophin is essential for the muscle, its absence causes an irreversible damage of this tissue, which is then replaced by adipose tissue [2]. Becker muscular dystrophy (BMD) is also a result of mutations in the *DMD* gene that do not completely disrupt the reading frame of the protein and allow for the production of a shorter version of a partially functional dystrophin [3].

A therapeutic strategy for DMD based on exon skipping has been developed, in which antisense oligonucleotides (aODNs) are used to mediate the elimination of the mutated exon, thus restoring the reading frame of the protein. In these conditions, the resulting synthesis of the dystrophin protein mimics the BMD phenotype [4,5,6,7,8,9]. Exon skipping could theoretically be used in 80–98% of DMD patients harboring deletions, duplications or nonsense mutations in the *DMD* gene [10]. There are currently four approved therapies of exon skipping for DMD, three by Sarepta Therapeutics and one by NS Pharma: Exondys 51, approved in 2016, for patients with a confirmed mutation in exon 51 (13% of all patients); Vyondys 53, approved in 2019, for patients with a confirmed mutation in exon 53 (8% of all patients); Amondys 45, approved in 2021, for the skipping of exon 45 (8% of all patients); and Vilepso, approved in 2020, designed to skip exon 53 on the DMD primary transcript (8–10% of all patients). Furthermore, researchers are currently working on new aODN-based drugs to promote the skipping of exons 44, 45, 50, 52, 53 and 55 of the *DMD* gene, either individually or in multiple exon skipping approaches [10,11,12]. 

Exon skipping based on antisense oligonucleotides is also being explored as a therapeutic strategy for other diseases such as mucolipidosis type II alpha/beta (ML II) [13] and titin-based dilated cardiomyopathy [14].

However, DMD therapies based on antisense oligonucleotides do not provide a permanent treatment for the disease, and nowadays, the clustered regularly interspaced short palindromic repeats CRISPR/Cas9 technology represents an alternative approach. In this direction, the removal of exon 23 and exons 21–23 in mdx mice has been reported [15,16,17]. Additionally, dystrophin expression has been restored in mice harboring a deletion of exon 44 and in dogs harboring a deletion of exon 50 [18,19].

PPRHs are non-modified DNA molecules formed by two mirror-repeat polypurine sequences in an antiparallel orientation linked by a pentathymidine loop that permits the establishment of intramolecular reverse-Hoogsteen bonds between both strands. These hairpins are able to bind by Watson and Crick bonds to their polypyrimidine targets in genomic DNA without losing their hairpin structure [20]. The binding of the PPRHs to their target polypyrimidine stretches causes then the displacement of the complementary polypurine strand of the DNA [20,21,22].

Depending on the DNA strand in which the polypyrimidine sequence is located, the corresponding PPRH targets either the template or the coding strand. Accordingly, PPRHs can act as antigens and also as antisense oligonucleotides against RNA if the polypyrimidine stretch is located in the coding strand of the DNA target. PPRHs were first described for gene silencing [23]. Next, we provided evidence that repair PPRHs, with a PPRH core and an extension tail on one of its ends, have the ability to correct different types of mutations in mammalian cells [24]. Thus, our technology could represent an additional strategy for gene therapy in those approaches aiming for the correction of point mutations responsible for genetic diseases.

To explore the capability of PPRHs to cause exon skipping at the DNA level which could be envisaged as a new therapeutic tool for genetic diseases such as DMD, we used a selectable marker gene as a model. In this regard, the cell model NB6 developed by Chen and Chasin [25] was selected since it contains an additional copy of exon 2 of the dihydrofolate reductase (*DHFR)* gene that causes a frameshift which abolishes DHFR activity in these cells. Editing PPRHs were designed to promote the skipping of this extra exon 2. The effects of these editing PPRHs upon transfection in NB6 cells and selection in a DHFR-selective medium were analyzed in individual colonies at the level of DNA, mRNA, protein and DHFR enzymatic activity.

## 2. Results

### 2.1. Design of Editing PPRHs

**Figure 1:**
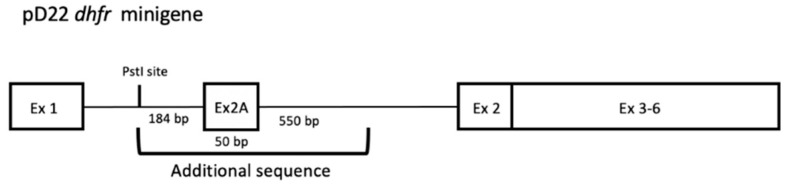
Organization of the pD22 *DHFR* minigene stably transfected in NB6 cells. A 0.8-kb PstI–BstEII genomic DNA fragment containing exon 2 and flanks of the Chinese hamster *DHFR* gene was cloned into the PstI site in intron 1 of pDCH1P to obtain the pD22 construct as described in Chen and Chasin [25].

The sequence corresponding to the pD22 *DHFR* minigene (Figure 1) was analyzed for the presence of polypurine target regions. 

**Figure 2:**
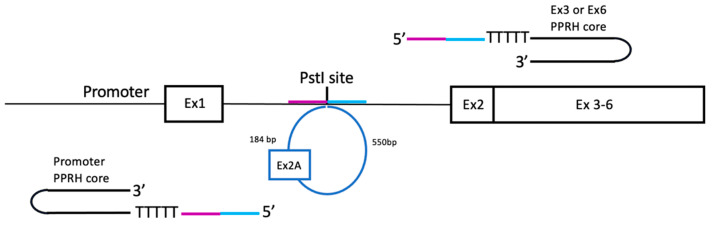
General approach for the design of the different editing PPRHs to edit the extra sequence (in light blue) including the additional exon 2 present in the *DHFR* minigene pD22. The complete structure for each editing PPRH contained a PPRH core and a sequence tail in its 5’ end homologous to 20 nt upstream (in purple) and/or 20 nt downstream (in blue) of the PstI restriction site present in the original pDCHIP minigene.

Three putative PPRH target regions were identified in the pD22 minigene, localized in the promoter, exon 3 and exon 6, respectively. These regions are the specific sequences where each PPRH core binds by means of their polypurine domains. Then, each PPRH core was extended at its 5’ end by a sequence tail homologous to 20 nt upstream and/or 20 nt downstream of the PstI restriction site present in the original *DHFR* minigene pDCH1P (Table 1 and Figure 2), which corresponded to the insertion site of the additional sequence including the extra exon 2. All editing PPRHs were designed following the long-distance approach described by Sole et al. [26], in which a pentathymidine sequence was included between the PPRH and the homologous tail. Our rationale was to provide a homologous recombination sequence in order to promote the recombination process and restore the wildtype sequence with just one copy of *DHFR* exon 2 and the original sequence of intron 1 (Figure 2). Depending on the location of the PPRH target with respect to the sequence to be edited, the editing tail was arranged to have the same sequence and orientation as the strand of the DNA to be recombined. 

As a negative control, sequence UDPstI corresponding only to the extension tail, 20 nt upstream and 20 nt downstream of the PstI restriction site present in the original *DHFR* minigene, but without a PPRH core attached, was used in the transfection experiments. 

### 2.2. DNA Sequencing in Edited Colonies

NB6 cells (30,000 to 150,000) were plated and transfected 24 hours later using 3 or 5 µg of editing PPRHs (117 or 195 nM, respectively, considering an average MW of 25,652 for the different editing PPRHs), either alone or in combination. Transfection of PPRHs carrying the whole homologous tail including both the 20 nt upstream the PstI site and the 20 nt downstream of this same site originated colonies in the DHFR-selective medium. The efficacy of the procedure ranged from 0.03 to 0.33%. When the homologous tail was provided in two separated regions by means of two different editing PPRHs, no colonies were obtained. Transfection of the sequence corresponding only to the homologous tail without a PPRH core only gave rise to a few colonies, less than 1% of those obtained with editing PPRHs. The combination of two different editing PPRHs both carrying a full-length homologous tail did not increase the frequency of edited colonies upon selection in the RPMI medium. 

**Figure 3:**
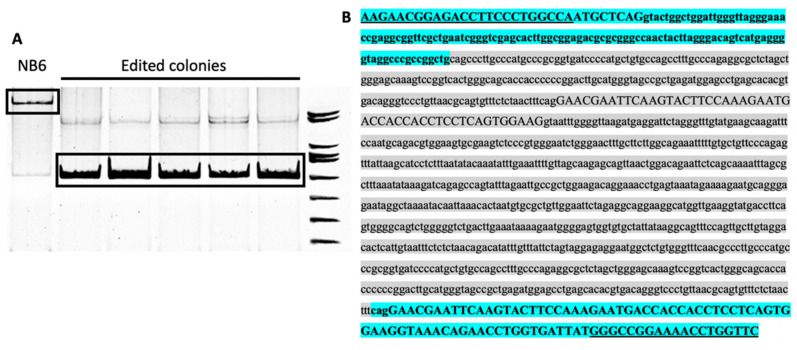
(**A**) PCR products from genomic DNA using a pair of primers in DHFR exon 1 and exon 3, respectively, obtained from either NB6 cells or random edited colonies, which were used in the sequencing reactions. (**B**) Highlighted in blue, the *DHFR* DNA sequence from exon 1 to exon 3 in the edited colonies compared to the original pD22 minigene, in which the extra sequence including the additional copy of exon 2 and its flanking regions is highlighted in grey. The sequences corresponding to the primers used to amplify the genomic DNA are underlined. Exons are indicated in uppercase whereas the intronic sequence is indicated in lowercase.

For each transfected editing PPRH, several cell colonies were individually expanded, genomic DNA was extracted and the region from *DHFR* exon 1 to exon 3 was PCR-amplified and sequenced. A negative control was performed with genomic DNA from *DHFR*-deficient DG44 cells, the recipient cell line for the initial transfection with the pD22 construct in order to establish the NB6 cell line. No band was obtained in these conditions. DNA sequencing results proved that all the analyzed colonies contained the wildtype *DHFR* sequence with only one copy of Exon 2 (Figure 3).

### 2.3. DHFR mRNA Expression in Edited Cell Colonies

**Figure 4:**
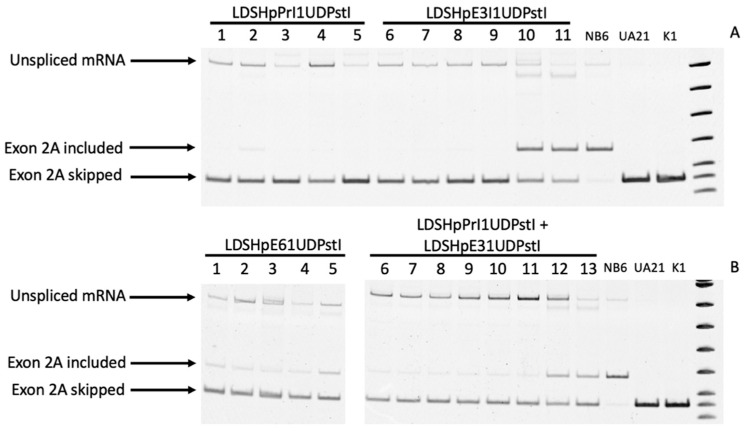
DHFR mRNA analysis in the edited clones. (**A**) Lanes 1 to 5, PCR products representative of random edited colonies from LDSHpPrI1UDPstI; lanes 6 to 11, PCR products representative of random edited colonies from LDSHpE3I1UDPstI. The species originating from NB6, UA21 and K1 cells (wildtype), respectively, correspond to the three last lanes. (**B**) Lanes 1 to 5, PCR products representative of random edited colonies from LDSHpE6I1UDPstI; lanes 6 to 13, PCR products representative of random edited colonies obtained with the combination of LDSHpPrI1UDPstI and LDSHpE3I1UDPstI. The species originating from NB6, UA21 and K1 cells (wildtype), respectively, are shown in the last three lanes.

Total RNA was extracted and RT-PCR-analyzed using a forward primer in exon 1 and a reverse primer in exon 3 to allow evaluating either the inclusion or skipping of the additional exon 2 according to the sizes of the PCR products, 166 vs 116 bp, respectively. As shown in Figure 4A,B, the mRNA species in NB6 cells revealed the presence of the additional exon 2, whereas in the edited colonies, the size of the amplified product corresponded to the skipping of this additional exon in accordance with the wildtype sequence present in UA21 cells carrying one copy of the endogenous *DHFR* gene and in K1 cells with two copies of the *DHFR* gene, both used as a positive control.

In the edited colonies obtained by transfection of LDSHpPRI1UDPstI, only the band corresponding to the skipping of the additional exon 2 was observed (Figure 4A, lanes 1 to 5), whereas in all the other approaches, the band corresponding to the inclusion of the additional exon 2 could still be detected in some colonies, although with a much lower intensity that in NB6 cells, in most of the cases (Figure 4A,B).

In all samples from the cells stably transfected with the pD22 minigene (NB6) and the corresponding edited colonies obtained upon transfection with the different editing PPRHs, we observed an amplified product whose size corresponded to unprocessed mRNA species due to intron 1 retention. These PCR products were sequenced, and the results revealed the presence of the full sequence for intron 1 and those for DHFR exons 1 and 2. This effect on mRNA processing was exclusive of transfected cells since the unprocessed species was not present in RNA samples from either UA21 or K1 cells.

**Figure 5:**
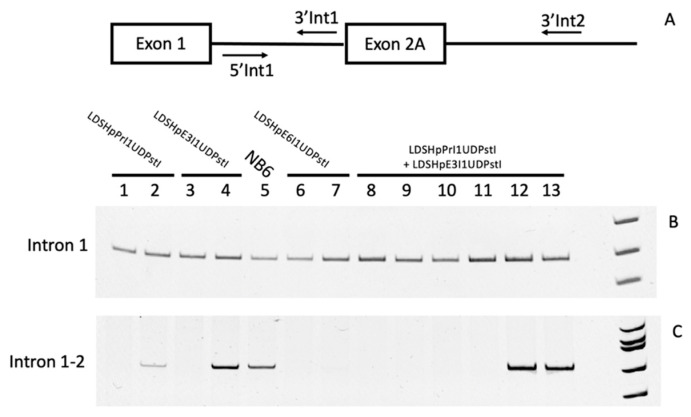
Intron retention in DHFR mRNA from edited clones. (**A**) Map of the locations of hybridization of the primers used in the analysis. (**B**) and (**C**) Lanes 1 and 2, PCR products representative of random edited colonies from LDSHpPrI1UDPstI; lanes 3 and 4, PCR products representative of random edited colonies from LDSHpE3I1UDPstI; lanes 6 and 7, PCR products representative of random edited colonies from LDSHpE6I1UDPstI; and lanes 8 to 13, PCR products representative of random edited colonies originating from the combination of LDSHpPrI1UDPstI and LDSHpE3I1UDPstI; lane NB6, species originating from NB6 cells.

In order to characterize all the mRNA species that could be detected in the parental cell line NB6 and the edited colonies, PCR reactions using a set of primers hybridizing either in intron 1 (Figure 5A,B) or in a combination pair for intron 1 and intron 2 (Figure 5A,C) were carried out. This approach confirmed intron 1 retention in all the samples and revealed the existence of unprocessed mRNA including the intron 2 region from the original *DHFR* minigene only in RNAs from either NB6 cells or those edited colonies that still produced DHFR mRNA, including the extra exon 2 copy. The different samples analyzed in Figure 4 and Figure 5 were as follows: lane 1 in Figure 4A corresponds to lane 1 in Figure 5B,C; lane 4 in Figure 4A corresponds to lane 2 in Figure 5B,C; lane 8 in Figure 4A corresponds to lane 3 in Figure 5B,C; lane 10 in Figure 4A corresponds to lane 4 in Figure 5A,C; lane 12 in Figure 4A corresponds to lane 5 in Figure 5B,C; lane 1 in Figure 4B corresponds to lane 6 in Figure 5B,C; lane 3 in Figure 4B corresponds to lane 6 in Figure 5B,C; lane 5 in Figure 4B corresponds to lane 7 in Figure 5B,C; lane 6 in Figure 4B corresponds to lane 8 in Figure 5B,C; lane 8 in Figure 4B corresponds to lane 9 in Figure 5B,C; lane 10 in Figure 4B corresponds to lane 10 in Figure 5B,C; lane 11 in Figure 4B corresponds to lane 11 in Figure 5B,C; lane 12 in Figure 4B corresponds to lane 12 in Figure 5B,C; and lane 13 in Figure 4B corresponds to lane 13 in Figure 5B,C.

### 2.4. DHFR Protein Levels and Enzymatic Activity in Edited Cell Colonies

**Figure 6:**
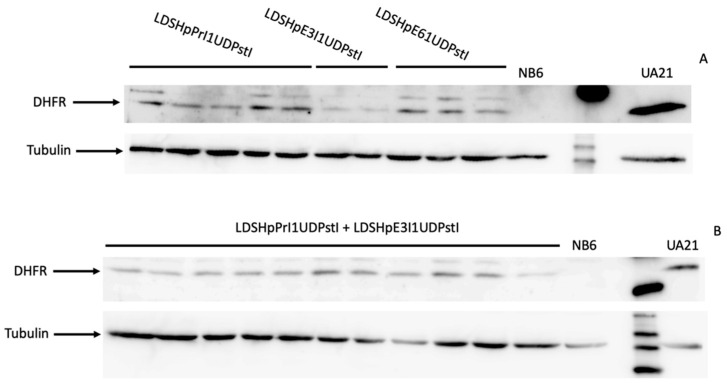
Levels of the DHFR protein. (**A**) Representative blot of the DHFR protein in total extracts from edited colonies obtained upon transfection with either LDHpPrI1UDPstI, LDHpE3I1UDPstI or LDHpE6I1UDPstI, NB6 or UA21 cells. (**B**) Representative blot of the DHFR protein in total extracts from edited colonies obtained upon transfection with the combination of LDHpPrI1UDPstI and LDHpE3I1UDPstI, NB6 or UA21 cells. Tubulin signal was used in all samples to normalize the results.

**Figure 7:**
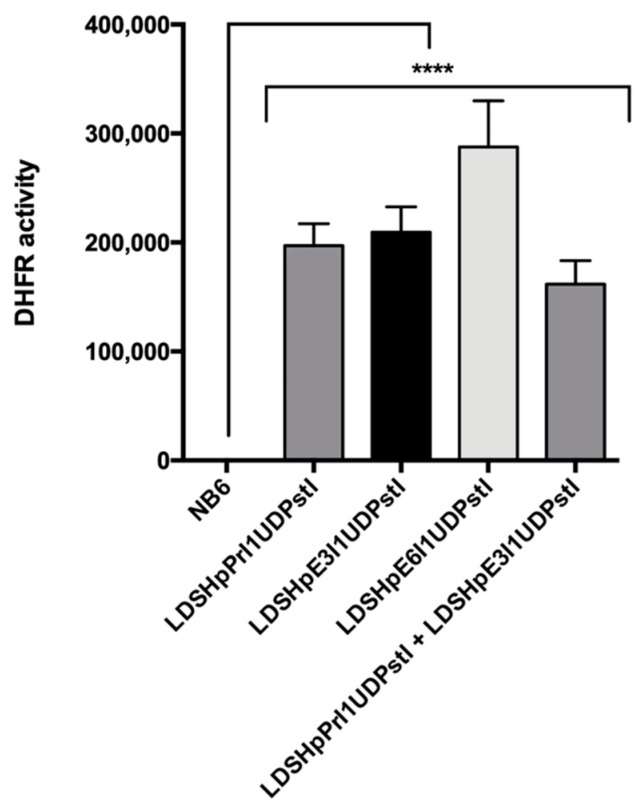
DHFR activity in control NB6 cells and random edited colonies obtained from transfections with the different editing PPRHs. Results are represented as the means ± SD values of three independent experiments. **** *p* < 0.0001.

Total extracts from the different edited colonies were analyzed by Western blotting to determine DHFR protein levels. As shown in Figure 6A,B, in all the clones analyzed, the DHFR protein was restored. Furthermore, the expressed protein showed high levels of DHFR activity in all colonies as determined in the enzymatic assay (Figure 7).

## 3. Discussion

In this work, we described the ability of the PPRH technology to cause exon skipping at the DNA level in a cell line model bearing an extra copy of *DHFR* exon 2 that leads to a frameshift. Previously, we showed that repair PPRHs were able to correct single-point mutations in two different mammalian genes, *DHFR* [26] and *APRT* [22], in their endogenous loci using a design in which the repair tail sequence contains only one nucleotide change with respect to the target region of the point mutation to be repaired. Here, we designed editing PPRHs following a similar approach, in which the PPRH core bears an extension tail homologous to 20 nt upstream and 20 nt downstream of a unique PstI restriction site that corresponds to the point of insertion of the additional sequence to be skipped. Thus, the editing tail included two separate regions that are apart in the parental DNA and that only come together when the skipping of the intervenient sequence takes place. In all the cases, we demonstrated the skipping of the extra *DHFR* exon 2 not only at the genomic level, but also at the mRNA level, which led to the translation of the functional DHFR protein.

We previously demonstrated effectiveness of the design of a PPRH core attached to an extension tail to correct point mutations and its generality of action as a repair PPRH technology [22,26]. Since one limitation of this design was a polypyrimidine target sequence in the vicinity of the mutation, we designed long-distance repair PPRHs by including an additional pentathymidine loop between the PPRH core and the repair tail when the target for the repair domain was located hundreds of nt upstream or downstream of the PPRH core. Long-distance repair PPRHs were able to correct their target mutations, indicating that adjacency between the PPRH core and the repair tail was not essential to correct the point mutation [22,26]. For the editing PPRHs, the same long-distance approach was used, and the three different PPRH cores located in either the promoter, exon 3 or exon 6 of the *DHFR* minigene were linked by a pentathymidine loop to the editing tail.

As can be seen in Figure 4, NB6 cells were able to correctly splice DHFR mRNA to a certain extent as previously reported when large numbers of NB6 cells were challenged in a DHFR-selective medium [25]. Chen and Chasin described the deletion of the duplicated sequence in pD22, possibly by intragenic homologous recombination, thus restoring the original structure of the pDCHIP1 minigene, and the appearance of point mutations at the splice sites [25]. Accordingly, we could observe a PCR product from NB6 genomic DNA with the same size as in the edited colonies, expanding from DHFR exon 1 to 3 (Figure 3A). However, in our conditions, these spontaneous revertants from NB6 cells were not able to neither express the DHFR protein nor display its enzymatic activity (Figure 6 and Figure 7). Nevertheless, this spontaneous deletion was associated with defective processing of DHFR pre-mRNA in NB6 cells, as shown by the levels of intron 1 retention in the corresponding mRNA molecules (Figure 4 and Figure 5). 

It is worth mentioning the same inefficient splicing of intron 1 in the pre-mRNA molecules in the edited colonies emerging from transfection with the different editing PPRHs, as shown in Figure 4 and Figure 5. We speculated that the defective splicing of DHFR pre-mRNA is inherent to the nature of the transfected cell model, since this inefficiency is not observed for the endogenous *DHFR* gene in UA21 and K1 cells, and that the amount of skipped and correct mRNA achieved upon transfection of the editing PPRHs could be higher in an endogenous gene setting. Nevertheless and despite inefficient processing of DHFR mRNA, we showed that skipping at the DNA level of the additional copy of *DHFR* exon 2 promoted by editing PPRHs led to the expression of the DHFR protein in these cells with its corresponding enzymatic activity.

Up until now, the main strategy to induce exon skipping has been based on antisense oligonucleotides. However, despite the encouraging results and the already available FDA-approved treatments for DMD, it is important to point out that exon skipping by antisense oligonucleotides requires their permanent delivery since this therapeutic approach works exclusively at the mRNA level. Repeated delivery of oligonucleotides is associated with toxicity and absorption by different tissues, mainly, liver and kidneys [27], Despite the safety profile of the current morpholino antisense oligonucleotides approved for DMD treatment, the FDA comments upon kidney toxicity, including potentially fatal glomerulonephritis that has been observed after administration of some antisense oligonucleotides and recommends monitoring kidney function in patients taking these drugs.

One advantage of the editing PPRH approach is that it promotes exon skipping at the DNA level and therefore does not require permanent treatment. On the other hand, we observed in a screening using PCR arrays for hepatic and renal toxicity that only a low percentage of genes were overexpressed upon transfection of PPRHs. These minor changes in gene expression did not cause any detrimental effect in HepG2 and 786-O human cell lines, respectively, as no toxicity in cell survival assays was observed [28].

Editing PPRHs could be envisaged as an alternative to genome editing technologies and, in particular, to CRISPR/Cas9. Although the frequency of skipping achieved by editing PPRHs is low, it does not require the generation of double-strand breaks. The precise correction of mutations through CRISPR-induced homologous directed repair (HDR) could be achieved, but the current approaches are limited since the frequency of HDR is much lower than that of NHEJ and implies the presence of Cas9, gRNA and donor DNA in the cells. The editing PPRH molecules combine all the elements needed in just one structure that targets a specific region of the DNA through the PPRH core and bears an extension tail that recombines with the original sequence in order to eliminate the intended exon. This structure allows for recombination since triplex-forming oligonucleotides stimulate recombination with donor DNAs depending on HDR [29,30] and transfection of a *Rad51* expression vector together with a repair PPRH increased 10-fold their frequency of correction [31], thus confirming that HDR participates in the correction process triggered by repair PPRHs. 

Several approaches to treat DMD by inducing NHEJ using the CRISPR/Cas9 technology to remove an internal DNA sequence or cause small indels have been described, such as the generation of double-strand breaks on both sides of the genomic region to be removed by a pair of gRNAs or the knockout of splicing sites of exons out of the frame to promote their skipping at the DNA level [32], following a strategy similar to aODN-based exon skipping. 

However, one of the main concerns with CRISPR/Cas9 is the possibility of small insertions, deletions or substitutions in the edited genome [33,34,35,36] that are usually the result of unspecific cuts of Cas9. On-target effects such as large deletions [37] and unexpected chromosomal truncations [38] have also been reported. In this regard, we determined by whole genome sequencing analyses the absence of any off-target effects in the genomic DNA from repaired cells upon transfection with repair PPRHs with a very similar structure to the present editing PPRHs. Neither unexpected indels caused by a repair PPRH were detected nor the insertion of the repair PPRH itself in any region of the genome [22]. 

In summary, this study represents a new application of the PPRH technology that provides further knowledge for their usage in genome editing for the treatment of monogenic diseases, either by the correction of point mutations or by exon skipping. Our future work will be aimed at exploring the feasibility of the exon skipping approach by editing PPRHs in endogenous gene loci. In this direction, in the absence of a possible metabolic selection for the corrected cells, next-generation sequencing at high coverage will be needed to evaluate the percentage of gene correction using the editing PPRH technology. In this way, the resulting sequences are aligned against the wildtype reference sequence and the percentage of correction or skipping is assessed with the Low Frequency Variant Detection algorithm. Since the efficacy of the editing procedure is low, next-generation sequencing will allow detecting this range of exon skipping using a depth of sequencing of 10,000× in those approaches without selection of the edited cells.

## 4. Materials and Methods

### 4.1. Oligonucleotides

PPRHs and primers were synthesized as non-modified oligodeoxynucleotides by Merck Life Science S.L.U. (Madrid, Spain). They were dissolved in a sterile RNase-free Tris-EDTA buffer (1 mM EDTA and 10 mM Tris, pH 8.0) at 10 µg/µL and stored at −20 °C until use. The Triplex-forming Oligonucleotide Target Sequence Search tool available at http://utw10685.utweb.utexas.edu/tfo/ was used to find the polypurine tracks present in the *DHFR* pD22 minigene and thus the polypyrimidine targets in order to design editing PPRHs. Table 1 describes all oligonucleotides names and sequences used in this work.

### 4.2. Cell Culture

Chinese hamster ovary cell lines DG44, NB6, UA21 and K1 were maintained at 37 °C in a humidified 5% CO2 atmosphere in a Ham’s F12 medium supplemented with 10% fetal bovine serum (both from GIBCO, Fisher Scientific S.L., Madrid, Spain). The cells were detached with 0.05% trypsin (Merck Life Science S.L.U., Madrid, Spain) for expansion and harvesting. To test for the capability of editing PPRHs to cause exon skipping at the DNA level, selection after transfection with the different PPRHs was performed in an RPMI 1640 medium supplemented with 7% dialyzed fetal bovine serum (both from GIBCO, Fisher Scientific S.L., Madrid, Spain), in which only the cells expressing the DHFR protein could survive.

### 4.3. Transfection of PPRHs and Selection

Transfection by editing PPRHs was carried out using 10 µM of the cationic liposome 1,2-Dioleoyl-3-trimethylammonium propane (DOTAP, Biontex, Munich, Germany) following the instructions of the manufacturer. A 200 µL mixture containing a serum-free medium, 10 µM DOTAP and 3–5 µg (117–195 nM) of the editing PPRHs was incubated for 20 min at RT and then added to the cells in 800 µL of a Ham’s F-12 medium containing 10% fetal bovine serum. After 24 h of incubation at 37 °C, to select the edited cells, the medium was changed to an RPMI 1640 medium supplemented with 7% dialyzed fetal bovine serum. Each transfection was performed at least three times, and for each successful editing PPRH, three to six colonies from each replicate were expanded.

### 4.4. DNA Sequencing 

The Wizard Genomic DNA Purification Kit (Promega, Madrid, Spain) was used to obtain total genomic DNA following the instructions of the manufacturer. The region between DHFR exons 1 and 3 was amplified by PCR using OneTaq DNA Polymerase (New England Biolabs, Ipswich, MA, USA). The primer sequences were 5’-AAGAACGGAGACCTTCCCTGGCCA-3’ in exon 1 and 5’-GAACCAGGTTTTCCGGCCCA-3’ in exon 3. Sequencing was carried out by Macrogen (Amsterdam, the Netherlands).

### 4.5. RNA Extraction and mRNA Analyses

Total RNA from edited cells was extracted using Trizol Reagent (Life Technologies, Madrid, Spain) following the instructions of the manufacturer. RNA concentration was quantified by measuring its absorbance (260 nm) in a Nanodrop ND-1000 spectrophotometer (Thermo Scientific, Wilmington, DE, USA). Complementary DNA was synthesized in 20 µL reaction mixture from 1 µg total RNA, 0.5 mM of each deoxyribonucleotide triphosphate (dNTP, Epicentre, Madison, WI, USA), 250 ng of random hexamers (Roche, Barcelona, Spain), 10 mM dithiothreitol, 200 units of a Moloney murine leukemia virus reverse transcriptase (RT), 20 units of an RNase inhibitor and 4 µL buffer (5×) (all three from Lucigen, Middleton, WI, USA). The reaction was incubated at 42 °C for 1 h. Then, PCR amplification was performed using 5 µL of the cDNA mixture. PCR reactions were typically carried out as described below. A standard 50 µL mixture contained 5 µL cDNA mixture, 10 µL 5× GC buffer, 0.2 mM dNTPs, 2.5 units of OneTaq (New England Biolabs, Ipswich, MA, USA) and 500 ng of each primer. The primers used were 5’-AAGAACGGAGACCTTCCCTGGCCA-3’ in DHFR exon 1 and 5’-GAACCAGGTTTTCCGGCCCA-3’ in DHFR exon 3. PCR cycling conditions were 3 min at 94 °C followed by 30 cycles of 30 s at 94 °C, 1 min at 61 °C and 1 min at 68 °C and a final extension step/stage of 5 min at 68 °C. A (-) RT reaction was also amplified for all RNA samples to rule out DNA contamination. PCR products were resolved in a 5% acrylamide gel in 1× TBE and the amplified bands were visualized using ethidium bromide staining.

To amplify the region corresponding to DHFR intron 1, the following primers were used: 5’-CCGAGGCGGTTCGCTGAATC-3’ and 5’-CCTGTCACGTGTGCTCAGGC-3’. In order to detect the additional sequence from intron 2 present in pD22, the primers used were 5’-CCGAGGCGGTTCGCTGAATC-3’ and 5’-TCCCACGGGAGACTTCGCACT-3’.

### 4.6. Western Blot Analyses 

Total extracts were prepared in a lysis buffer (50 mM HEPES ((4-(2-hydroxyethyl)-1-piperazineethanesulfonic acid), 0.5 M NaCl, 1.5 mM MgCl_2_, 1 mM EGTA (ethylene glycol-bis(β-aminoethyl ether)-N,N,N′,N′-tetraacetic acid), 10% glycerol, 1% Triton X-100, pH 7.2) supplemented with Protease Inhibitor Mixture (Merck Life Science S.L.U., Madrid, Spain) from the cells harvested by trypsinization. The samples were maintained at 4 °C on ice for 1 h with vortexing every 15 min. Cell debris was removed by centrifugation (10,000× *g* for 10 min) and the total protein concentration was determined in the supernatants using a BioRad protein assay (based on the Bradford method, using bovine serum albumin as a standard (Merck Life Science S.L.U., Madrid, Spain).

Total cell extracts (100 µg) were electrophoresed on SDS–12% polyacrylamide gels and transferred to a polyvinylidene difluoride membrane (Immobilon P, Millipore, Madrid, Spain) using a semidry electroblotter. Blots were incubated with antibodies against hamster DHFR (1:200 dilution; Pocono Rabbit Farm & Laboratory, Canadensis, PA, USA), and tubulin (1:100 dilution; CP06, Calbiochem, Merck, Darmstadt, Germany) to normalize the results. The signals were detected with HRP-conjugated anti-rabbit antibodies (1:2500 dilution; P0399, Dako, Denmark) for DHFR or with anti-mouse antibodies (1:2500 dilution; sc-2005, Santa Cruz Biotechnology, Heidelberg, Germany) for tubulin and enhanced chemiluminescence using ECL^TM^ Prime Western Blotting Detection Reagent (GE Healthcare, Barcelona, Spain). Chemiluminescence was detected with Image- Quant LAS 4000 Mini (GE Healthcare, Barcelona, Spain).

### 4.7. DHFR Activity 

The method to determine DHFR activity is based on the incorporation of radioactive deoxyuridine into DNA. DHFR catalyzes transformation of the folate supplied in the medium to tetrahydrofolate, a cofactor needed for the reductive methylation of deoxyuridylate to thymidylate. Thymidylate is then incorporated into DNA, which is isolated by precipitation [39]. Six-well dishes were plated with either 10,000 NB6 or edited cells in 1 mL RPMI medium. The next day, 2 µCi 6-[3H] deoxyuridine (20 mCi/mmol, Moravek Biochemicals, Inc., USA) was added for 24 h. After two washes with phosphate-buffered saline, the cells were lysed in 100 µL of 0.1% sodium dodecyl sulfate. The lysate was spotted onto 31ET paper (Whatman, Merck Life Science S.L.U., Madrid, Spain), washed three times in 66% cold ethanol containing 250 mM NaCl, dried and counted in a scintillation counter.

### 4.8. Statistical Analyses

All transfections were performed at least in triplicates, and for each experiment, at least five independent colonies were analyzed for each editing PPRH. For DHFR activity, the data are represented as the means ± SD values of three independent experiments. Statistical analyses were performed using ordinary one-way ANOVA with Dunnett’s multiple comparison tests. Analyses and representation of the data were carried out using the GraphPad Prism version 6.0 software (GraphPad, La Jolla, CA, USA).

## Figures and Tables

**Table 1:** Design of the editing PPRHs to skip the additional *DHFR* exon 2 in pD22 stably transfected in NB6 cells. The name, sequence and location of the target for each editing PPRH are indicated. The sequence highlighted in purple corresponds to the 20 nt upstream of the PstI restriction site, whereas the sequence highlighted in blue corresponds to the 20 nt downstream of this same site; both referred to the original sequence of the wildtype minigene.

Name	Editing PPRH Sequence	Location of the Target for the PPRH Core
LDSHpPrI1UPstI	5’CTGCAGCCGGCGGGCCTACCTTTTTGGGGAAGGAAAAGTGGGTGACATTTTTACAGTGGGTGAAAAGGAAGGGG3’	Promoter
LDSHpPrI1UDstI	5’ACCGCGGGCATGGGCAAGGGCTGCAGCCGGCGGGCCTACCTTTTTGGGGAAGGAAAAGTGGGTGACATTTTTACAGTGGGTGAAAAGGAAGGGG3’	Promoter
LDSHpE6I1DPstI	5’CCCTTGCCCATGCCCGCGGTTTTTTAGGAGGAAAAAGGCATCAAGTTTTTGAACTACGGAAAAAGGAGGA3’	Exon 6
LDSHpE6I1UDPstI	5’GGTAGGCCCGCCGGCTGCAGCCCTTGCCCATGCCCGCGGTTTTTTAGGAGGAAAAAGGCATCAAGTTTTTGAACTACGGAAAAAGGAGGA3’	Exon 6
LDSHpE3I1UDPstI	5’GGTAGGCCCGCCGGCTGCAGCCCTTGCCCATGCCCGCGGTTTTTTGGACCAAGAGGTAAGGATTTTTAGGAATGGAGAACCAGG3’	Exon 3
UDPst1	5’GGTAGGCCCGCCGGCTGCAGCCCTTGCCCATGCCCGCGGT3’	None

## Data Availability

Not applicable.
